# Robust Carbonated Structural Color Barcodes with Ultralow Ontology Fluorescence as Biomimic Culture Platform

**DOI:** 10.34133/2021/9851609

**Published:** 2021-05-04

**Authors:** Panmiao Liu, Zhongde Mu, Muhuo Ji, Xiaojiang Liu, Hanwen Gu, Yi Peng, Jianjun Yang, Zhuoying Xie, Fuyin Zheng

**Affiliations:** ^1^Department of Anesthesiology, Pain and Perioperative Medicine, The First Affiliated Hospital of Zhengzhou University, Zhengzhou, China 450052; ^2^Jiangsu Cancer Hospital & Jiangsu Institute of Cancer Research & The Affiliated Cancer Hospital of Nanjing Medical University, Nanjing 210009, China; ^3^Department of Anesthesiology, The Second Affiliated Hospital, Nanjing Medical University, Nanjing, China; ^4^State Key Laboratory of Bioelectronics, School of Biological Science and Medical Engineering, Southeast University, Nanjing, China 210096; ^5^Key Laboratory for Biomechanics and Mechanobiology, Beijing Advanced Innovation Center for Biomedical Engineering, School of Biological Science and Medical Engineering, Beihang University, Beijing 100083, China

## Abstract

Photonic crystal (PC) barcodes are a new type of spectrum-encoding microcarriers used in multiplex high-throughput bioassays, such as broad analysis of biomarkers for clinical diagnosis, gene expression, and cell culture. Unfortunately, most of these existing PC barcodes suffered from undesired features, including difficult spectrum-signal acquisition, weak mechanical strength, and high ontology fluorescence, which limited their development to real applications. To address these limitations, we report a new type of structural color-encoded PC barcodes. The barcodes are fabricated by the assembly of monodisperse polydopamine- (PDA-) coated silica (PDA@SiO_2_) nanoparticles using a droplet-based microfluidic technique and followed by pyrolysis of PDA@SiO_2_ (C@SiO_2_) barcodes. Because of the templated carbonization of adhesive PDA, the prepared C@SiO_2_ PC beads were endowed with simultaneous easy-to-identify structural color, high mechanical strength, and ultralow ontology fluorescence. We demonstrated that the structural colored C@SiO_2_ barcodes not only maintained a high structural stability and good biocompatibility during the coculturing with fibroblasts and tumor cells capture but also achieved an enhanced fluorescent-reading signal-to-noise ratio in the fluorescence-reading detection. These features make the C@SiO_2_ PC barcodes versatile for expansive application in fluorescence-reading-based multibioassays.

## 1. Introduction

Multiplex assay has achieved great progress in detection and quantification of a broad variety of analytes in diverse practical applications, especially in biomedical-related fields [1–3]. Due to high flexibility, fast detection, and good repeatability, suspension arrays are attracting increasing attention in the multiplex analysis [4–6]. Among numerous different suspension arrays, photonic crystal (PC) beads encoded suspension arrays that are well used in view of their excellent encoding stability and biocompatibility [7, 8]. Benefiting from the spherical nanoplatform and stable characteristic reflection peak, PC barcodes have recently achieved significant developments in multiplex three-dimensional (3D) scale analysis, including cell capture [9–11], bacteria analysis [12], drug screening [13], and bioassays [14–17]. Unfortunately, most of the as-reported PC barcodes still cannot be transformed from laboratory samples to practical products due to some of their limited features. First, PC beads were always encoded by the reflection spectrum; thus, the encoded information only existed at the spherical vertex based on the Bragg diffraction mechanism [18]. The spectrum acquisition would be very difficult when the beads are small enough according to the current spectrum measurement techniques. Second, the current PC barcodes have been widely reported in the applications of fluorescence-reading-based detection [19–21], while still performing negative impact on the detection sensitivity or reliability due to their strong ontology fluorescence leading to undesired background interference. Finally, those PC barcodes working as cell-culturing and capture carriers are easy to be broken or endocytosed by surface cultured cells because of low adhesion between internally assembled particles, thus leading to cell apoptosis or cytometaplasia [14, 22]. Thus, the development of a novel type of PC barcodes with desired features and distinct advantages is still required.

Polydopamine (PDA) has been reported to observably improve the color visibility of structural color materials (PC or amorphous structure), and it is a common melanin-like material prepared by autoxidation of dopamine [23–26]. PDA possesses abundant catechol functional groups on its surface and also exhibits an unparalleled adhesion to most surfaces, even those with low interfacial energy, such as mussel adhesion protein [27–29]. The more outstanding performance of PDA is that its strong adhesion can be sustained or improved after the carbonization via thermal treatment [30–32]. Therefore, the integrated strategy by combining PC barcodes and carbonization of adhesive PDA is a promising approach to achieve high mechanical strength and low ontology fluorescence.

In this paper, we present a new type of carbon-bonded high-strength structural colored PC barcodes with desired capabilities; they simultaneously perform easy-to-identify structural color, high mechanical strength, and ultralow ontology fluorescence, as well as good biocompatibility for cell culturing and capture and multiple analysis. The barcodes are fabricated by the assembly of monodispersed PDA-coated silica (PDA@SiO_2_) nanoparticles with a droplet-based microfluidic technique and followed by pyrolysis of PDA@SiO_2_ (C@SiO_2_) barcodes. Owing to the transformation of PDA to carbon by pyrolysis treatment, the structural colored C@SiO_2_ PC beads exhibit easy-to-identify structural color and prominent improvement on mechanical strength and ontology fluorescence. Results showed that the ontology fluorescence of C@SiO_2_ PC beads only accounts for 0.14 to 0.37 parts of SiO_2_ PC beads. And the mechanical strength is 365% of the SiO_2_ PC beads, 190% for PDA@SiO_2_ PC beads, and even higher than that of conventional SiO_2_ beads. More attractively, C@SiO_2_ PC beads performed an improved signal-to-noise ratio by an average of 2.5 times than the conventional SiO_2_ PC beads after staining of fluorescence makers. These C@SiO_2_ barcodes demonstrate excellent biocompatibility with the characterization of cell activity and morphology, and their encoding remains constant with both high mechanical stability and color visibility during multiple events of cell capture and cell culturing at the surface. These features make this novel type of barcodes an ideal platform for biological multivariate analysis, multicellular suspension culture, and multiple types of cell capture and multiorgans-on-barcodes.

## 2. Results and Discussion

### 2.1. Fabrication of Barcodes

**Figure 1:**
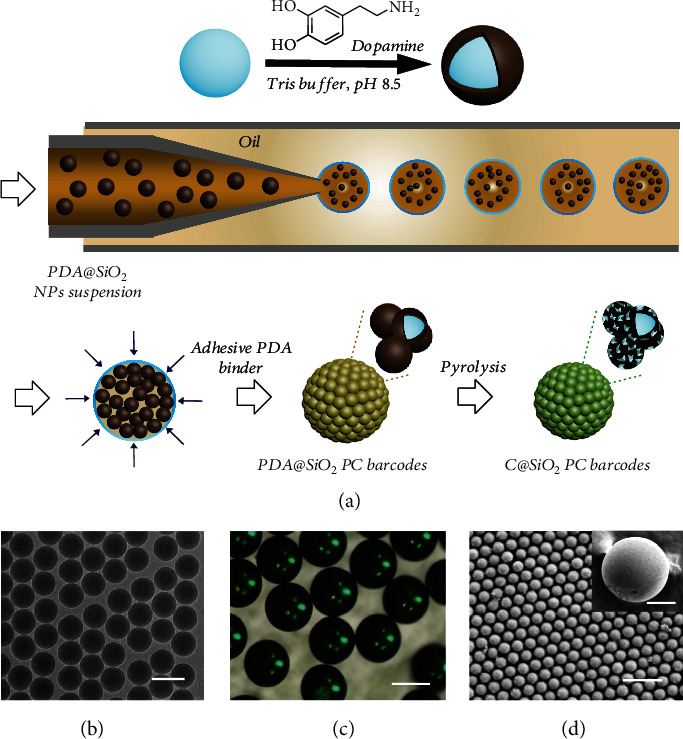
(a) Schematic diagram of the fabrication of C@SiO_2_ PC beads. (b) TEM image of the prepared PDA@SiO_2_ particles with 252 nm particle sizes. The insert scale bar is 300 nm. (c) Optical microscope photograph of C@SiO_2_ PC beads. The insert scale bar is 300 *μ*m. (d) FESEM image of the appearance (i) and surface (ii) of C@SiO_2_ beads. The insert scale bar is 100 *μ*m for insert image, 1 *μ*m for (d).

In a typical experiment, C@SiO_2_ PC beads were fabricated by the evaporation of droplet templates containing monodisperse PDA@SiO_2_ nanoparticles followed by pyrolysis. The detailed fabrication for C@SiO_2_ PC beads is shown in Figure 1(a). Firstly, PDA@SiO_2_ particles were obtained by polymerization dopamine forming a PDA shell on the uniform SiO_2_ particles in 10 mM tris-buffer. Then, PDA@SiO_2_ particles were assembled into spherical PC microspheres (PDA@SiO_2_ PC beads) with a droplet-based microfluidic technique. Finally, C@SiO_2_ PC beads were obtained after pyrolysis treatment of PDA@SiO_2_ PC beads in the absence of oxygen. In the microfluidic system, the PDA@SiO_2_ particles could directly assemble into the stable PC beads without any extra treatment due to the PDA binder properly around each building block. Figure 1(b) shows the transmission electron microscope (TEM) image of the prepared PDA@SiO_2_ particles with 252 nm particle sizes. These building block-PDA@SiO_2_ nanoparticles with thin-coated PDA shell presented an admirable spherical shape and good monodispersity, which showed a brilliant red color after centrifugation (Figure S1). The standard spherical PDA@SiO_2_ PC beads displaying a homogeneous slightly yellowish structural color were obtained after drying the droplet containing PDA@SiO_2_ nanoparticles (Figure S2). Followed by pyrolysis, the C@SiO_2_ PC beads, as shown in Figure 1(c), were obtained and exhibited a more visible and luminous green color under natural light, superior to the SiO_2_ PC beads (Figure S3). The nanoparticles of the barcode beads mainly formed spherical assemblies with a regular arrangement and ultimately performed as a close-packed colloidal crystal array structure in the sphere (Figure 1(d)). The barcode beads were derived from microfluidic droplets that contained monodispersed silica nanoparticles, and their initial size and subsequent variations after being PDA-coated and carbonized could be customized from several to hundreds of micrometers. The adjustable preparation strategies are performed not only by varying the flow rates of the water and oil phases but also by using different concentrations of the nanoparticles for droplet generation. Here, barcode particles with a diameter of about 300 *μ*m were generated for the functional and morphological characterization as cell-culturing and capture carriers.

### 2.2. Optical Principle and Structural Color Encoding

**Figure 2:**
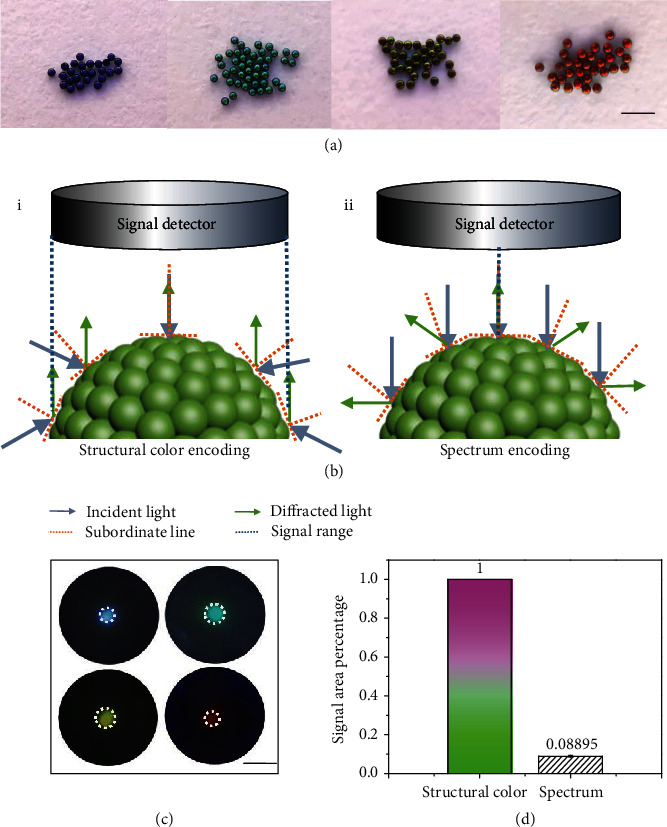
(a) Super-close-focus photographs of C@SiO_2_ beads with four classes of structural colors. Insert scale bar is 1 mm. (b) Schematic diagram of PC beads encoded by structural color (i) and spectrum (ii). (c) Optical photographs of C@SiO_2_ beads with four classes of structural colors. The insert scale bar is 300 *μ*m. (d) The comparison of the signal area between structural color mode and spectral mode of PC beads.

Based on the optical principle of PC, the central diffraction wavelength (structural color) of the C@SiO_2_ PC beads is estimated by Bragg's equation under normal incidence [18],

(1) $$\begin{array}{c} \lambda =1.633\times d\times n_{a}, \end{array}$$

where *λ* is the central diffraction wavelength, *d* is the center-to-center distance between two nanoparticles, and *n*_*a*_ is the average refractive index of C@SiO_2_ beads. By changing the diameters of the C@SiO_2_ beads, four classes of colored C@SiO_2_ beads were obtained (Figures 2(a) and S4). These beads displayed visible colors under natural light that can be clearly distinguished by the naked eyes. The signal capture of the visible color is schemed in Figure 2(b), (i). Since the incident light may be acquired from various directions, the color signal range of the PC microspheres can be basically equal to their volume. Nevertheless, the spectrum signal is usually obtained by detecting the diffraction spectrum via injecting incident light perpendicular to the vertex tangent of the microspheres (Figure 2(b), (ii)). This means that the detected signal comes from only one point of the microspheres. The optical photographs in Figure 2(c) also demonstrated that the spectral signal is just one point located on the top of the spheres. By contrast, the signal area of the spectral mode only accounts for 0.08 parts of the structural color mode (Figure 2(d)). These results indicated that the structural color-encoded beads have a significant advantage on the signal acquisition than the spectrum encoded for PC barcodes.

### 2.3. Mechanical Strength and Stability

**Figure 3:**
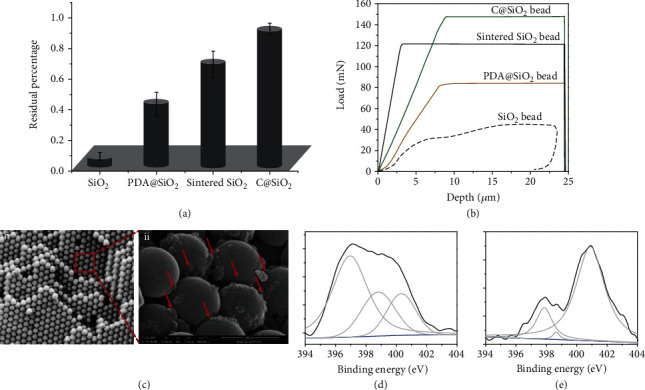
(a) Column chart of ultrasonic treatment to SiO_2_ beads, PDA@SiO_2_ beads, sintered SiO_2_ beads, and C@SiO_2_ beads. (b) Compression test curves of SiO_2_ beads, PDA@SiO_2_ beads, sintered SiO_2_ beads, and C@SiO_2_ beads. (c) Cross-section and zoomed detailed images of C@SiO_2_ PC beads. Insert bar is 1 *μ*m for (i) and 300 nm for (ii). (Red arrows are the positions of C granules.) (d, e) High-resolution XPS narrow scans of the N1s region of PDA (d) and py-PDA (e). N1s peak reveals graphitic N at 400.9 eV, pyrrolic N at 399.0 eV, and pyridinic N at 398.4 eV.

Mechanical strength and stability are the main factors to be considered in the application of barcode beads, especially in cell culture and capture. The highly color visible C@SiO_2_ PC beads are converted by pyrolysis of coating PDA, which plays a role as an adhesive polymer and increases the PC beads' binding strength. As expected, the mechanical properties, i.e., compression resistance and ultrasonic damage resistance, increased upon PDA to C conversion. The compression tests were implemented by NanoTest, which were to test the load stresses of beads to reach 25 *μ*m depths (Figures S5-S8).  Figure 3(a) shows the resulted curves of load vs. depth for SiO_2_ PC beads, PDA@SiO_2_ PC beads, sintered SiO_2_ PC beads, and C@SiO_2_ PC beads. The PDA@SiO_2_ PC beads endured nearly 82 ± 5 mN stresses before breaking, which were double of the SiO_2_ PC beads. After pyrolysis, the endured pressure increased, with values of 149 ± 2 mN for C@SiO_2_ PC beads and 120 ± 15 mN for sintered SiO_2_ PC beads. Furthermore, the residual rate after suffering two minutes of ultrasonic treatment increased after pyrolysis. As shown in Figure 3(b), the average residual rate was calculated to be 0.12 for SiO_2_ PC beads, 0.48 for PDA@SiO_2_ PC beads, 0.59 for sintered SiO_2_ PC beads, and 0.95 for C@SiO_2_ PC beads. As a result, compared to the initial SiO_2_ PC beads, the C@SiO_2_ PC beads exhibited remarkably increased compression resistance (365%) and ultrasonic damage resistance (792%). In order to understand the reasons for the increase of mechanical strength for C@SiO_2_ PC beads, the section-cross of the above beads were investigated by the field emission scanning electron microscopy (FESEM), as shown in Figures 3(c) and S9. Except for SiO_2_ PC beads, many grains were observed between the particles of PDA@SiO_2_ PC beads, sintered SiO_2_ PC beads, and C@SiO_2_ PC beads. Raman spectra of SiO_2_ PC beads, PDA@SiO_2_ PC beads, and C@SiO_2_ PC beads confirmed that the grains of PDA@SiO_2_ PC beads could be contributed to a self-adhesion reaction of PDA shells, the grains for sintered SiO_2_ PC beads might be the fusion of SiO_2_ particles by pyrolysis, and the grains for C@SiO_2_ PC beads may be due to the pyrolysis of PDA shells (Figure S10). Regardless of the fact that grains existed in all these PC beads, C@SiO_2_ PC beads own the strongest mechanical strength. This may be attributed to the graphitic structure which was formed by pyrolysis of PDA, which is more stable than that of the PDA supermolecule and even stronger than the silicon-oxygen bond between the sintered SiO_2_ PC beads. XPS was further used to analyze the chemical composition and confirm this speculation. The peaks at 284.8, 401.1, and 532.1 eV correspond to C1s, N1s, and O1s in the PDA@SiO_2_ beads and C@SiO_2_ beads (Figure S11), respectively. After pyrolysis, a significant decrease in the N1s and O1s peaks was observed due to chemical reduction and graphitization [33]. High-resolution N1s and C1s spectra from the C@SiO_2_ beads revealed significant differences in the carbon chemistry of the PDA. The high-resolution N1s spectra indicated the presence of graphitic N at 400.9 eV, pyrrolic N at 399.0 eV, and pyridinic N at 398.4 eV (Figures 3(d) and 3(e)). The major N1s peak observed in the PDA@SiO_2_ beads corresponded to pyrrolic N (57.55%), whereas the N1s peak observed in the C@SiO_2_ beads corresponded to graphitic N (82.77%). The high-resolution C1s spectra exhibited similar shifts after pyrolysis. The high-resolution C1s spectra indicated the presence of C-H bonding at 281.3 eV, C-C bonding at 284.5 eV, C-O bonding at 285.5 eV, C-N sp^2^ bonding at 286.1 eV, C-O-C bonding at 286.8 eV, and C-N sp^3^ bonding at 288.2 eV (Figure S12). The C-H level decreased significantly after pyrolysis, from 15.58% (PDA@SiO_2_) to 3.17% (C@SiO_2_). Moreover, the level of C-O bonding increased from 2.26% (PDA@SiO_2_) to 32.68% (C@SiO_2_). As a result, the strong graphitic interaction and additional interaction area induced by template pyrolysis PDA endow the C@SiO_2_ PC beads with a strong mechanical strength, which brings great convenience to the real application of PC barcode beads.

### 2.4. Ultralow Ontology Fluorescence Properties

**Figure 4:**
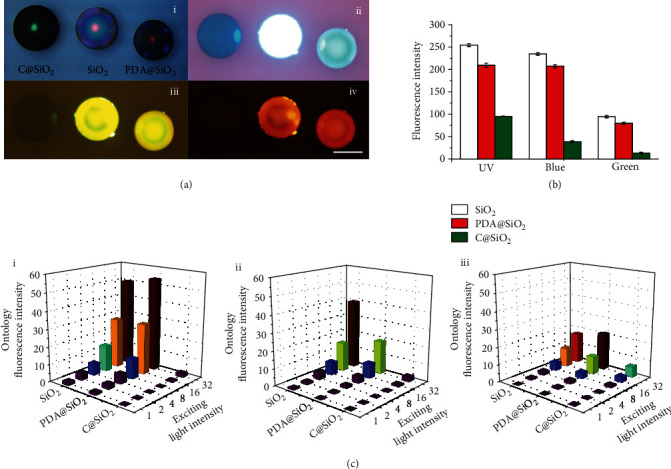
(a) The optical photographs of sintered SiO_2_ beads, PDA@SiO_2_ beads, and C@SiO_2_ beads excited by natural (i), UV (ii), blue (iii), and green (iv) light, respectively. The insert bar is 200 *μ*m. (b) The ontology fluorescence intensity of SiO_2_ beads, PDA@SiO_2_ beads, and C@SiO_2_ beads shot by UV, blue, and green light, respectively. (c) The ontology fluorescence intensity of SiO_2_ beads, PDA@SiO_2_ beads, and C@SiO_2_ beads shot by different exciting intensities of UV (i), blue (ii), and green (iii) light.

The development of PC barcodes should be devoted to gain a low ontology fluorescence to improve the detection signal-to-noise ratio in fluorescence-reading applications. To demonstrate the fluorescent property of C@SiO_2_ PC beads, the sintered SiO_2_ PC beads, PDA@SiO_2_ PC beads, and C@SiO_2_ PC beads were compared excited by different light (Figure 4(a)). As shown in Figure 4(b), the fluorescence intensity of C@SiO_2_ PC beads is 0.36 of the sintered SiO_2_ PC beads under ultraviolet (UV) light (EX: 361~385 nm), 0.25 under blue light (EX: 465~495 nm), and 0.20 under green light (EX: 540~580 nm). Adjusting the exciting light intensity to different degrees, the ultralow ontology fluorescence of C@SiO_2_ PC beads remains unchanged. Obviously, the ultralow fluorescence intensity of C@SiO_2_ PC beads is more remarkable when the exciting light tends to a short light (Figure 4(c) and Figure S13). Based on the above structure analysis on C@SiO_2_ beads, the ultralow ontology fluorescence of C@SiO_2_ PC beads may result from pyrolysis eliminating the organic small molecules which could produce fluorescence in the original PDA@SiO_2_ beads, thereby reducing the ontology fluorescence of the C@SiO_2_ beads [34, 35]. Furthermore, despite the fact that the addition of PDA can reduce the ontology fluorescence of PDA@SiO_2_ PC beads, the extent of the reduction is very limited, only about 0.80 of the sintered SiO_2_ PC beads. Thus, the advantage of ultralow ontology fluorescence may allow C@SiO_2_ PC barcodes to improve the detection sensitivity through depressing the ontology signal interference.

### 2.5. Biocompatibility Characterization of Barcodes

**Figure 5:**
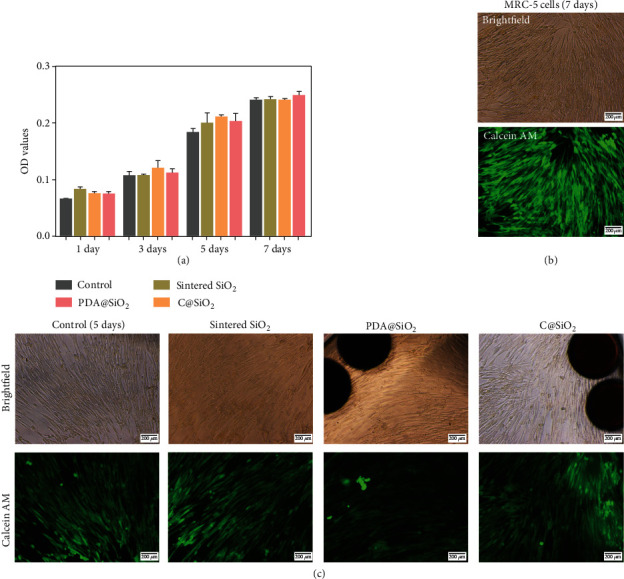
The cytotoxicity of the barcodes. (a) MTT assay of the attachment and proliferation viability by coculturing with MRC-5 cells. (b) IFM micrographs of MRC-5 cells coculturing with C@SiO_2_ barcodes for 7 days in TCP. (c) IFM micrographs of MRC-5 cells in TCP and coculturing with sintered SiO_2_ beads, PDA@SiO_2_ beads, and C@SiO_2_ beads for 5 days in TCP.

Achieving both high mechanical stability and color visibility, it is important to ensure the biocompatibility of the developed C@SiO_2_ PC barcodes for further biomedical applications [9, 13]. The cytocompatibility of C@SiO_2_ PC barcodes was assessed by quantitative analysis of cell proliferation activity by MTT assay and CCK-8 tests and qualitative analysis of morphology from fluorescence micrographs by coculturing with human embryonic lung fibroblasts (MRC-5) cells and comparison with the control, sintered SiO_2_ PC beads, and PDA@SiO_2_ PC beads. The final OD values in direct proportion to the numbers of viable MRC-5 cells are given in Figure 5(a), which reveals the viability of MRC-5 cells cocultured with control, sintered SiO2 PC beads, PDA@SiO_2_ PC beads, and C@SiO_2_ PC barcodes. The OD value of MRC-5 cells cocultured with each kind of beads showed a significant increase from day 1 to day 7, indicating that cell proliferation activity increased sharply in a manner similar to growth on native extracellular matrix or tissue culture plates (TCPs). Compared with the cell viability on day 5, the OD value on day 7 did not have a significant increase, suggesting the saturation of cells on TCPs in the same culture medium. Furthermore, cell proliferation assays demonstrated that the contained SiO_2_ nanoparticles, PDA, and carbonated PDA did not release any chemical pigment resulting in acute cytotoxicity and did not significantly influence cell viability. Before a typical cell experiment, CCK-8 tests have also demonstrated that the C@SiO_2_ barcodes present excellent biocompatibility coculturing with MRC-5 (Figure S18).

The biocompatibility of the C@SiO_2_ PC barcodes was also further confirmed by observing the morphology of cells cocultured with them for 5 and 7 days via an inverted fluorescence microscope (IFM) (Figures 5(b) and 5(c)). The viable cells can be visually observed after calcein AM staining with green fluorescence. MRC-5 cells have spindle morphology and polarity with long axis direction. There will be some vacancies among cells for contact inhibition when the long axis direction of cells has contact with each other after quick extension and proliferation as shown in Figure 5(b). Meanwhile, this morphology of MRC-5 cells proliferated coculture with sintered SiO_2_ beads, PDA@SiO_2_ beads, and C@SiO_2_ beads similar to TCPs as shown in Figure 5(c), indicating that these barcodes have excellent cytocompatibility to promote cell proliferation. Furthermore, cell density showing a significant increase when cultured for 5 days further verified the accuracy of the MTT cell proliferation assay. Therefore, barcodes possessed excellent biocompatibility with improved proliferation viability and good phenotypic shape of MRC-5 cells, which should find various promising applications in the fields of fluorescence-reading-based multibioassays.

### 2.6. Multivariate Analysis Platform and Cell Capture

**Figure 6:**
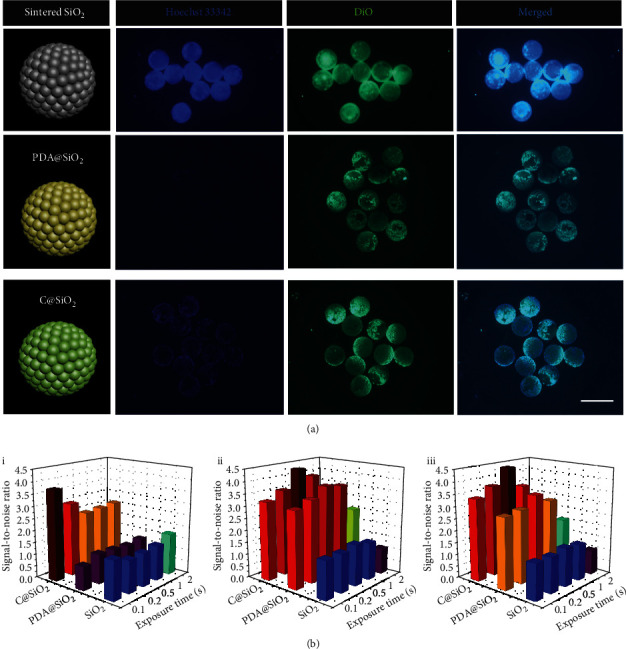
(a) Fluorescence microscopy images of Hoechst 33342 staining cell nucleus, DiO staining cell membrane, and merged images of Hoechst 33342 and DiO for the captured 5-8F cells on the PLL-coated sintered SiO_2_ beads, PDA@SiO_2_ beads, and C@SiO_2_ beads, respectively. The insert bar is 500 *μ*m. (b) Different exposure-time-obtained fluorescence signal-to-noise ratios between Hoechst 33342 staining cell nucleus (i), DiO staining cell membrane (ii), and merged images of Hoechst 33342 and DiO (iii) of the captured 5-8F cells with the PLL-coated sintered SiO_2_ beads, PDA@SiO_2_ beads, and C@SiO_2_ beads, respectively.

To test the superiority of C@SiO_2_ beads' usability, sintered SiO_2_ PC beads, PDA@SiO_2_ PC beads, and C@SiO_2_ PC beads were implemented to capture 5-8F cells, which were labeled by corresponding fluorescent probes that gave blue and green fluorescence to the nucleus and cell membrane, respectively.  Figure 6(a) exhibited the fluorescent microscopy images of the staining cell nucleus (Hoechst 33342), staining cell membrane (DiO), and merged images of Hoechst 33342 and DiO of SiO_2_ PC beads, PDA@SiO_2_ PC beads, and C@SiO_2_ PC beads. These images stated that C@SiO_2_ carriers showed an ultralow ontology fluorescence, which could even be completely ignored, compared with SiO_2_ PC beads.  Figure 6(b) and Figures S14-S16 further revealed different exposure-time-obtained fluorescence signal-to-noise ratios between Hoechst 33342 staining cell nucleus (i), DiO staining cell membrane (ii), and merged images of Hoechst 33342 and DiO (iii) of the captured 5-8F cells with the PLL-coated sintered SiO_2_ beads, PDA@SiO_2_ beads, and C@SiO_2_ beads, respectively. The results showed that C@SiO_2_ beads are available to eliminate the interference of ontology fluorescence and improved the signal-to-noise ratio by an average of 2.5 times than the sintered SiO_2_ PC beads (Figure S17). Notably, although PDA@SiO_2_ also was capable of depressing ontology fluorescence and thus increased the signal-to-noise ratio to some extent, the increased capacity was not ineffective for the Hoechst 33342 staining cell nucleus. The discrepancy may result from the transformation of PDA to C-PDA. The phenolic hydroxyl group and amino group in PDA have a strong electron ability, which promotes the efficiency of energy transfer and/or electron transfer when PDA@SiO_2_ beads contact with fluorescent molecules. But after pyrolysis, these electron-withdrawing groups are destroyed, and the ability to quench the fluorescence of C@SiO_2_ beads disappears.

**Figure 7:**
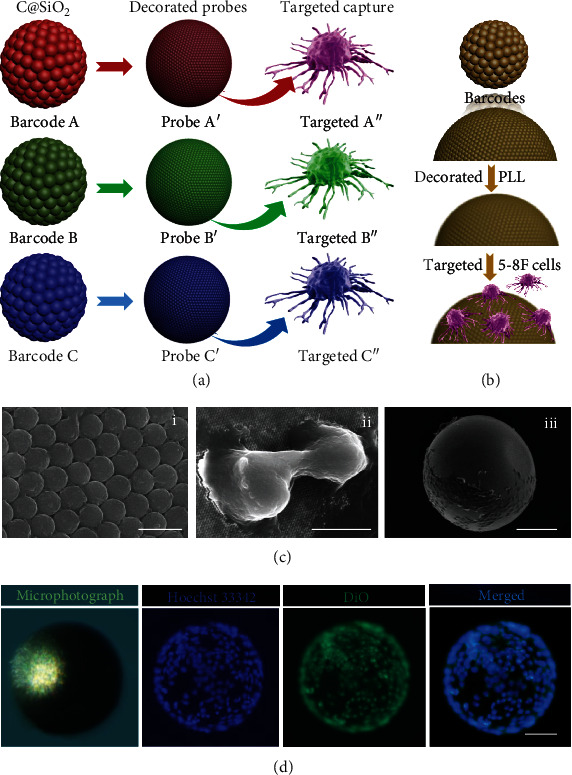
(a) Schematic of the C@SiO_2_ barcodes in capturing and detecting multiple types of cells; three types of barcode particles that exhibit red (A), green (B), and blue (C) structural colors were modified with three types of targeted probes (A', B', C') and multitarget red (A”), (B”), and (C”) fluorescence-stained cells, respectively. (b) Schematic of the C@SiO_2_ beads used for capturing 5-8F cells; the surface of the beads is decorated with PLL molecular. (c) FESEM images of PLL-coated C@SiO_2_ beads after cell culture for six days: (i) surface particles, (ii) captured 5-8F cell, and (iii) the appearance. The insert scale bar is 500 nm for (i), 5 *μ*m for (ii), and 100 *μ*m for (iii). (d) Microphotograph and fluorescence microscopy images of C@SiO_2_ beads with captured 5-8F cells, including Hoechst 33342 for staining cell nucleus, DiO for staining cell membrane, and merged images of Hoechst 33342 and DiO. The insert scale bar is 100 *μ*m.

Cell analysis plays a key role in the biomedical diagnosis [36–38]. In the real case of biomedical diagnosis, analysis for only one kind of cells is not sufficient to diagnose a specific disease from the extraordinary complexity of biological specimens [39, 40]. According to the unique optical encoding properties, sintered SiO_2_ PC beads have served as cell capture carriers for the capture of various circulating tumor cells. Moreover, the surface of the barcode beads with a spherical periodic array topography further realized cell analysis and release for multiple types of circulating tumor cells simultaneously [9]. C@SiO_2_ PC barcodes employ the characteristic reflection peaks as their distinct encoded elements and their peak positions are based on the periodic structure or refractive index of the materials and remain constant during cell capture, adhesion, and culture on their surface; this indicates the high encoding accuracy of the microcarriers in multiplexing [6, 17]. C@SiO_2_ PC barcodes provide a nanopatterned surface topography with ordered hexagonal symmetry of the nanoparticles and reduce steric hindrance and increased density of the biomaterial molecules, such as protein antibodies (e.g., antiepithelial cell-adhesion molecule) or nucleotide probes (e.g., DNA aptamers) [9, 10]. They are free to interact with some specific surface proteins of multiple cells and, thus, increase the efficiency of the cell adhesion and targeted capture. An important requirement for using barcode particles for multiple cell capture and detection is the accurate identification of their coded information during the entire process of cell capture. To demonstrate the reliability of the barcode particles in capturing and detecting multiple types of cells, three types of barcode particles that exhibit red (A), green (B), and blue (C) structural colors were modified with three types of targeted probes (A', B', C'), respectively. These targeted probes modified barcode particles which were then mixed and incubated in a multitarget solution of red fluorescence-stained cells (A”), green fluorescence-stained cells (B”), and blue fluorescence-stained cells (C”) (Figure 7(a)). Because of the specific binding between the targeted probes and their corresponding target cell types, we expected to observe the specific cells on the surface of the barcode particles when their corresponding targeted probes were present.

The C@SiO_2_ PC beads were placed in a system of cultured human nasopharyngeal carcinoma (5-8F) cells. Because of the small number of chemical groups on the C@SiO_2_ surface that enable cell adhesion, the practical capture density of cells on the barcode beads is limited (Figure S19). To solve this problem, polylysine (PLL), widely used to promote cell adhesion, was modified on the surface of the beads [41, 42]. The process of 5-8F cells captured on the surface of C@SiO_2_ PC beads is illustrated in Figure 7(b). The C@SiO_2_ barcode beads were decorated by PLL molecular, and thus, they not only provided a large surface area and close-packed hemispheric array surface topography for cell capture but also provided a specific procell adhesion platform to increase the efficiency of cell capture. As shown in Figure 7(c), (i), the surface of the C@SiO_2_ beads after being modified by PLL was still arranged with a hexagonal close packing. Taking into account this spherical array topography, 5-8F cells could successfully adhere to as well as extend to the whole surface of the sphere (Figure 7(c), (ii, iii)). To confirm the cell-captured capability, 5-8F cells were cultured for 6 days in the cultured medium mixed with PLL decorated C@SiO_2_ carriers. The everyday condition of cells on the surface of beads was observed by a light microscope. As shown in Figure S20, with the growth of cultured time, the 5-8F cells gradually increased on the beads and even stacked with each other to cover the whole sphere to form a film with a thickness of up to 32 *μ*m. More importantly, the shape of the C@SiO_2_ beads remained unchanged for six days in the complex cell culture medium, indicating that the high stability of the beads is enough for a wider range of applications, superior to PDA@SiO_2_ beads (Figure S21). The optical microphotograph in Figure 7(d) revealed that C@SiO_2_ beads covered with thick 5-8F cells still maintained their initial brilliant yellow color. The corresponding fluorescent microscopy images of the cell-captured beads with a staining cell nucleus (Hoechst 33342), staining cell membrane (DiO), and merged images of Hoechst 33342 and DiO were shown subsequently. These fluorescent microscopy images exhibited that the ontology fluorescence of the C@SiO_2_ carriers was basically negligible, so that the fluorescent information of the surface cells can be read out without consideration of background interference (Figure S22). Therefore, the C@SiO_2_ carriers simplified the extraction of cellular fluorescence signal by eliminating the interference of carriers' fluorescence during the analysis of cellular information. All these results demonstrated that the C@SiO_2_ PC beads not only could maintain well both high mechanical stability and color visibility during cell capture and culture but also performed an excellent ability on fluorescent-reading after staining by fluorescence makers.

## 3. Conclusion

In summary, we developed a new type of structural color-encoded PC barcodes. These colored PC barcodes are fabricated by the assembly of monodispersed PDA@SiO_2_ nanoparticles with a droplet-based microfluidic technique and followed by pyrolysis. Owing to the transformation of PDA to carbon by pyrolysis treatment, the as-prepared C@SiO_2_ PC beads performed bright high-visible structural colors and simultaneous prominent improvement on mechanical strength and ontology fluorescence. More importantly, C@SiO_2_ PC beads performed an improved signal-to-noise ratio in fluorescence-based detection. These C@SiO_2_ barcodes demonstrate excellent biocompatibility with the characterization of cell activity and morphology, and their encoding remains constant with both high mechanical stability and color visibility during multiple events of cell capture and cell culturing at the surface. These features make the C@SiO_2_ PC barcodes ideal for extensive application in fluorescence-reading-based multibioassays. We believe the proposed C@SiO_2_ PC barcodes will provide a span-new platform for multiplex analysis, especially in biological multivariate analysis, multicellular suspension culture, and multiple types of cell capture and multiorgans-on-barcodes.

## 4. Materials and Methods

### 4.1. Materials

3-Hydroxytyramine hydrochloride (DA·HCl), tris(hydroxymethyl)aminomethane (Tris), and hexadecane were purchased from Aladdin (Shanghai, China). SiO_2_ particles with different diameters were purchased from Nanjing Nanorainbow Biotechnology Co., Ltd. (China). Hoechst 33342 and 3,3-dioctadecyloxacarbocyanine perchlorate (DiO) were purchased from Shanghai Beyotime Biotechnology Co. Ltd. (Shanghai, China). The human NPC 5-8F cell line was provided by the Research Center of Clinical Oncology of the Affiliated Jiangsu Cancer Hospital (Nanjing Medical University, Nanjing, China). Polylysine (PLL) and fetal bovine serum without mycoplasma were purchased from Tianhang Biological Technology Co., Ltd., Zhejiang. PBS (pH = 7.4) was laboratory homemade. Dimethyl sulfoxide (DMSO) was purchased from Sigma (USA). Deionized water (18.0 M*Ω* cm, Milli-Q Gradient System, Millipore) was used in all experiments. All chemical reagents were used without further purification.

### 4.2. Preparation of PDA@SiO_2_ Particles

PDA@SiO_2_ was prepared by dispersing 0.1 g SiO_2_ particles into 10 mL DA and Tris solution (pH = 8.5) for stirring for 18 h. Then, the obtained particles were cleaned by centrifugalization for three times and further purification with a filter membrane.

### 4.3. Preparation of C@SiO_2_ Beads

Firstly, the particles of PDA@SiO_2_ were homogeneously dispersed in water, with the particle quality fraction of 2%. Subsequently, the particle solution was used as the dispersed phase, and hexadecane with 1% Hypermer 2296 was used as the continuous phase. Then, when the hexadecane and aqueous suspension were simultaneously injected into the PTFE tube, the aqueous suspension was broken into droplets by the hexadecane flows at the needle tip. The hexadecane took the suspension droplets into the collection container which was filled with hexadecane with 2% Hypermer 2296 and maintained heated at 75°C, and then, solid PDA@SiO_2_ beads were derived and then were thoroughly washed with hexane to remove the hexadecane. At last, the PDA@SiO_2_ beads were pyrolyzed at 400°C for 8 hours with nitrogen protection.

### 4.4. MTT Assay for Cell Viability

The human MRC-5 cells were cultured in DMEM (Corning, Manassas, VA, USA) supplemented with 5% FBS (Gibco, Grand Island, USA) in the presence of 5% CO_2_ at 37°C. The assay was carried out in a 96-well plate. The barcodes were sterilized with 75% alcohol and washed twice with PBS and then incubated in medium overnight. The number of cells in each well was 1 × 10^4^. After MRC-5 cell seeding, the 96-well plate was placed in the incubator for 24 h at 37°C and 5% CO_2_. Then, we transferred the substrates to a new 96-well plate to get rid of the cells adhering on the plate, and new medium and 20 *μ*L of MTT (98%) solution in PBS (5 mg mL^−1^) were added into each well and incubated at 37°C for 4 h. The culture medium in each well was then removed, and 200 *μ*L of dimethyl sulfoxide (DMSO, Sigma, USA) was added to completely dissolve the formazan crystals formed in the cells on the substrates. The absorbance of each well at 490 nm was measured by a microplate reader (Synergy HT, BioTek, USA).

### 4.5. Fluorescence Staining for Cell Viability

We used fluorescence staining to detect the cell viability of MRC-5 cells. Calcein AM (molecular prober, USA) is a kind of dye, which can penetrate cells and be used for determining the vitality of most eukaryotic cells. In living cells, a nonfluorescent calcein AM will convert to green fluorescent calcein after the intracellular esterase hydrolyzes acetoxymethylester. We incubated the MRC-5 cells cocultured with barcodes in 10 *μ*M calcein AM solution for 20 min in an incubator. Finally, the stained MRC-5 cells were rinsed in PBS and observed by fluorescence microscope (Olympus IX71, Olympus, Japan).

### 4.6. PLL-Coated C@SiO_2_ Beads

Polylysine with concentration of 0.01% was prepared in a PBS solution. C@SiO_2_ beads were firstly washed with ethanol (once) and PBS (three times). Then, the washed beads were immersed in the 0.01% PLL solution at room temperature. After 24 hours, the excess liquid was wiped off, and C@SiO_2_ beads were washed repeatedly by PBS solution for three times with a sterile operation.

### 4.7. Cell Culture

The human NPC cell line (5-8F) was cultured in RPMI-1640 (Corning, Manassas, VA, USA) supplemented with 5% FBS (Gibco, Grand Island, USA) in the presence of 5% CO_2_ at 37°C. Before the experiment, SiO_2_, PDA@SiO_2_, and C@SiO_2_ beads needed to be rinsed once with absolute ethanol and three times with PBS and soaked in 0.1 mg/mL polylysine (PLL) overnight. Then, digested cells in a logarithmic growth phase with trypsin were resuspended slightly, and the resuspended cells (3 mL, 1 × 10^5^ cells per mL) were added to new dishes which contained PLL-coated SiO_2_, PDA@SiO_2_, or C@SiO_2_ beads. In the following 6 days, photos were taken with the light microscope to evaluate the mechanical strength. Meanwhile, in order to compare the ontology fluorescence of the beads and label these captured cells, a part of the beads with cells cultured to day 5 was selected, and the cell membrane and nucleus were stained with DiO (Beyotime, China) and Hoechst 33342 (Beyotime, China), respectively, and fluorescence photos were then obtained by a fluorescence microscope.

### 4.8. Damage-Resistant Experiment

Ultrasonic damage test was conducted in the bottom of the ultrasonic instrument with thirty SiO_2_ beads, PDA@SiO_2_ beads, sintered SiO_2_ beads, and C@SiO_2_ beads soaked in a tube with 2 mL water. After two minutes of ultrasonic treatment, the final residual bead numbers (the integrated spheroidal was regarded as one bead number) were counted by the metalloscope (Olympus BX51). The compression test was performed by the Nantes with set-up parameters (maximum load was 150 mM, loading rate was 0.3 mN/s, and unloading rate is 5 mN/s). The beads lined up under the crosshair of the multiple-objective microscope and then were compressed by a 100 *μ*m radius spherical diamond probe (Figure S4).

### 4.9. Characterization

The SiO_2_@PDA images were obtained by Transmission Electron Microscopy (TEM, JEM2100F). The images of appearance, surface, and cross-section of C@SiO_2_ beads and the cells on C@SiO_2_ beads were obtained by a Field Emission Scanning Electron Microscope (FESEM, Zeiss Ultra Plus). The surface element analysis of beads was implemented by X-ray photoelectron spectroscopy (XPS, GB-T19500-2004). The Raman spectra were measured using an inVia Renishaw Raman microscope system (Renishaw, New Mills, UK). The microphotographs of the beads were taken by the metalloscope (Olympus BX51) with a CCD camera (Media Cybernetics Evolution MP 5.0). The absolute reflectance of PC beads was measured by a spectrometer (QE65000, Ocean Optics) with light source (DH-2000UV-VIS-NIR, Mikropack) and optical fiber (QR200-7-UV-BX, Ocean Optics) using a diffuse reflection standard plate (WR-D97-30, Oceanhood) as a completely diffuse reflector. 1931 CIE coordinates were obtained from absolute reflectance calculated by a specific software. Fluorescence images of DiO, Hoechst 33342, and merged DiO and Hoechst 33342 were measured by an inverted fluorescence microscope (Olympus, MVC10). The cells on the beads for FESEM were pretreated with a graded series of concentrations of ethanol (0%, 25%, 50%, 75%, and 100%).

## Data Availability

All other data are available from the corresponding authors upon reasonable request.

## Supplementary Materials

Supplementary Materials Figure S1: zoomed TEM image of the prepared PDA@SiO_2_ particles with a thin PDA shell. Figure S2: optical microscope photograph of PDA@SiO_2_ PC beads. Figure S3: optical microscope photograph of SiO_2_ PC beads. Figure S4: photograph of the four classes of colored C@SiO_2_ beads under natural light. Figure S5: schematic of compression test for PC beads. Figure S6: photograph of SiO_2_ PC beads, PDA@SiO_2_ PC beads, C@SiO_2_ PC beads, and sintered SiO_2_ PC beads adhered on the sample stage of the NanoTest system. Figure S7: typical load vs. depth curve analysis showing the compression (breaking) of the PC beads measured by the NanoTest system. Figure S8: optical images of PDA@SiO_2_ PC beads before and after compression on the NanoTest system. Figure S9: magnified FESEM images of cross-section of the SiO_2_ beads, PDA@SiO_2_ beads, sintered SiO_2_ beads, and C@SiO_2_ beads. Figure S10: Raman spectra of SiO_2_ beads, PDA@SiO_2_ beads, and C@SiO_2_ beads. Figure S11: XPS spectra of PDA@SiO_2_ PC beads and C@SiO_2_ PC beads. Figure S12: high-resolution XPS narrow scans of the N1s region of PDA and py-PDA. Figure S13: the ontology fluorescence intensity of SiO_2_ beads, PDA@SiO_2_ beads, and C@SiO_2_ beads shot by UV, blue, green, and natural light under exciting light intensity with different degrees from 1 to 32. Figure S14: different exposure-time-obtained fluorescence images of Hoechst 33342 staining cell nucleus of the captured 5-8F cells with the PLL-coated sintered SiO_2_ beads, PDA@SiO_2_ beads, and C@SiO_2_ beads. Figure S15: different exposure-time-obtained fluorescence images of the DiO staining cell membrane of the captured 5-8F cells with the PLL-coated sintered SiO_2_ beads, PDA@SiO_2_ beads, and C@SiO_2_ beads. Figure S16: different exposure-time-obtained fluorescence images of merged images of Hoechst 33342 and DiO of the captured 5-8F cells with the PLL-coated sintered SiO_2_ beads, PDA@SiO_2_ beads, and C@SiO_2_ beads. Figure S17: the ratio of signal recognition ability between C@SiO_2_ barcodes and the sintered SiO_2_ PC barcodes. Figure S18: CCK-8 results of C@SiO_2_ barcodes culturing with MRC-5. Figure S19: optical microscope photograph of non-PLL-coated C@SiO_2_ beads culturing with 5-8F cells on the second day. Figure S20: microscopy images and zoomed images of PLL-coated C@SiO_2_ beads culturing with 5-8F cells from 1 to 6 days. Figure S21: the fluorescent intensity of background, beads, and surface cells in Hoechst 33342, DiO, and merged Hoechst 33342 and DiO fluorescent-reading images of the C@SiO_2_ bead. Figure S22: optical microscopy images of PLL-coated PDA@SiO_2_ beads and C@SiO_2_ beads culturing with 5-8F cells on the sixth day. Figure S23: the reflectance spectrum of SiO_2_ beads (solid line) and sintered SiO_2_ beads (dotted line).
