# Supplementary material for: Robust Carbonated Structural Color Barcodes with Ultralow Ontology Fluorescence as Biomimic Culture Platform
Source: Research (Wash D C). 2021 May 4;2021:9851609. doi: 10.34133/2021/9851609 (PMC8118130; doi:10.34133/2021/9851609)
Supplement: Supplementary Materials — Figure S1: zoomed TEM image of the prepared PDA@SiO2 particles with a thin PDA shell. Figure S2: optical microscope photograph of PDA@SiO2 PC beads. Figure S3: optical microscope photograph of SiO2 PC beads. Figure S4: photograph of the four classes of colored C@SiO2 beads under natural light. Figure S5: schematic of compression test for PC beads. Figure S6: photograph of SiO2 PC beads, PDA@SiO2 PC beads, C@SiO2 PC beads, and sintered SiO2 PC beads adhered on the sample stage of the NanoTest system. Figure S7: typical load vs. depth curve analysis showing the compression (breaking) of the PC beads measured by the NanoTest system. Figure S8: optical images of PDA@SiO2 PC beads before and after compression on the NanoTest system. Figure S9: magnified FESEM images of cross-section of the SiO2 beads, PDA@SiO2 beads, sintered SiO2 beads, and C@SiO2 beads. Figure S10: Raman spectra of SiO2 beads, PDA@SiO2 beads, and C@SiO2 beads. Figure S11: XPS spectra of PDA@SiO2 PC beads and C@SiO2 PC beads. Figure S12: high-resolution XPS narrow scans of the N1s region of PDA and py-PDA. Figure S13: the ontology fluorescence intensity of SiO2 beads, PDA@SiO2 beads, and C@SiO2 beads shot by UV, blue, green, and natural light under exciting light intensity with different degrees from 1 to 32. Figure S14: different exposure-time-obtained fluorescence images of Hoechst 33342 staining cell nucleus of the captured 5-8F cells with the PLL-coated sintered SiO2 beads, PDA@SiO2 beads, and C@SiO2 beads. Figure S15: different exposure-time-obtained fluorescence images of the DiO staining cell membrane of the captured 5-8F cells with the PLL-coated sintered SiO2 beads, PDA@SiO2 beads, and C@SiO2 beads. Figure S16: different exposure-time-obtained fluorescence images of merged images of Hoechst 33342 and DiO of the captured 5-8F cells with the PLL-coated sintered SiO2 beads, PDA@SiO2 beads, and C@SiO2 beads. Figure S17: the ratio of signal recognition ability between C@SiO2 barcodes and the sintere [file 9851609.f1.doc]

Robust Carbonated Structural Color Barcodes with Ultra-low Ontology Fluorescence as Biomimic Culture Platform

**Panmiao Liu^1^, Zhongde Mu^3^, Muhuo Ji^5^, Xiaojiang Liu^2^, Hanwen Gu^1^, Yi Peng^3^, Jianjun Yang^1^*, Zhuoying Xie^2^*, and Fuyin Zheng^4^***

*^1^Department of Anesthesiology, Pain and Perioperative Medicine, The First Affiliated Hospital of Zhengzhou University, Zhengzhou, China, 450052*

*^2^State Key Laboratory of Bioelectronics, School of Biological Science and Medical Engineering, Southeast University, Nanjing, China, 210096*

*^3^Jiangsu Cancer Hospital & Jiangsu Institute of Cancer Research & The Affiliated Cancer Hospital of Nanjing Medical University, Nanjing 210009, P. R. China*

*^4^Key Laboratory for Biomechanics and Mechanobiology, Beijing Advanced Innovation Center for Biomedical Engineering, School of Biological Science and Medical Engineering, Beihang University, Beijing, 100083, China*

*^5^Department of Anesthesiology, The Second Affiliated Hospital, Nanjing Medical University, Nanjing, China*

Correspondence should be addressed to Jianjun Yang; jianjunyang1971@163.com, Zhuoying Xie; zyxie@seu.edu.cn or Fuyin Zheng; zhengfuyin@buaa.edu.cn


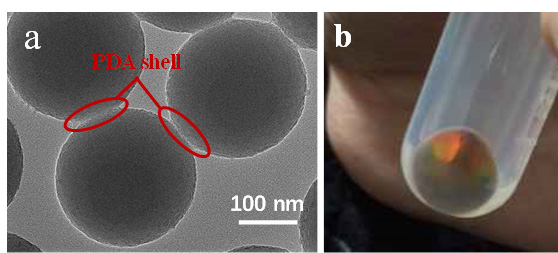


**Figure S1.** (a) Zoomed TEM image of the prepared PDA@SiO_2_ particles with a thin PDA shell. (b) Photograph of sediment after centrifugation of PDA@SiO_2_ particles.


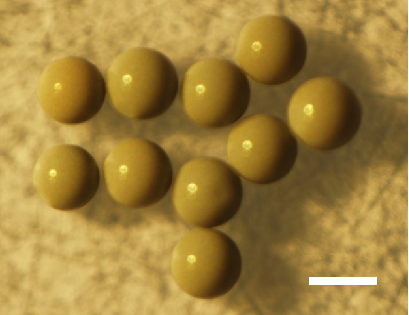


**Figure S2.**  Optical microscope photograph of PDA@SiO_2_ PC beads. Insert bar is 200 µm.


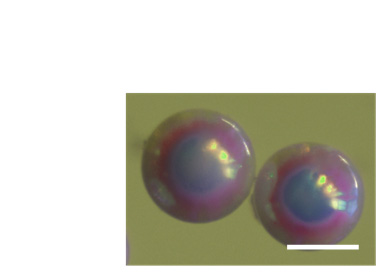


**Figure S3.** Optical microscope photograph of SiO_2_ PC beads. Insert bar is 200 µm.


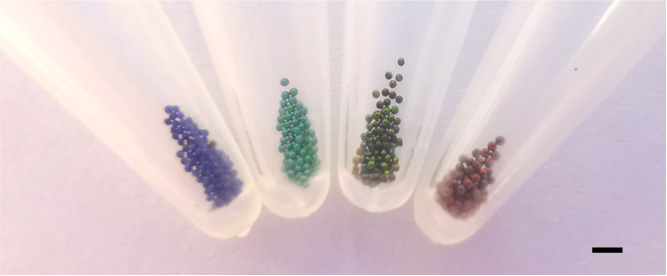


**Figure S4.** Photograph of the four classes of colored C@SiO_2_ beads under natural light. Insert bar is 1 mm.


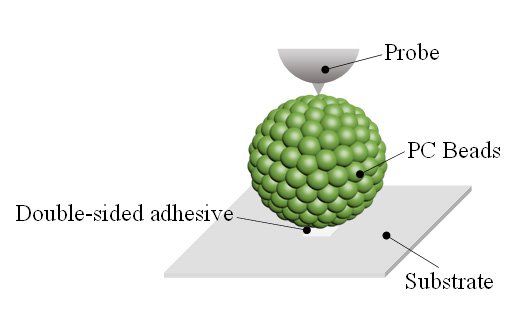


**Figure S5.** Schematic of compression test for PC beads.


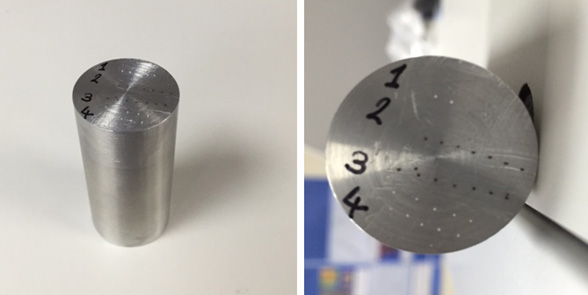


**Figure S6.** Photograph of SiO_2_ PC beads (1), PDA@SiO_2_ PC beads (2), C@SiO_2_ PC beads (3), and sintered SiO_2_ PC beads (4) adhered on the sample stage of NanoTest system.


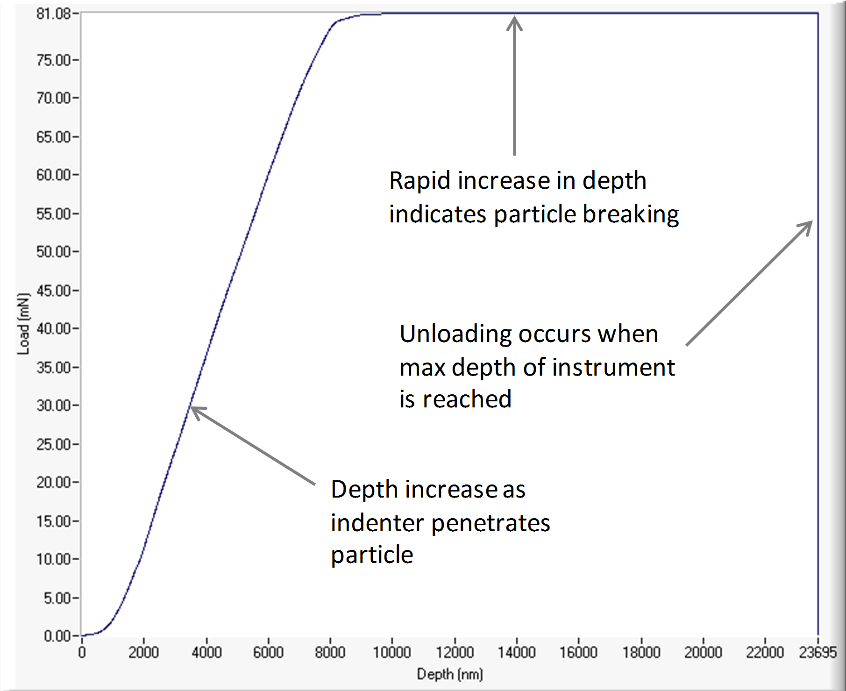


**Figure S7.** Typical load vs depth curve analysis showing the compression (breaking) of the PC beads mearsured by NanoTest system.


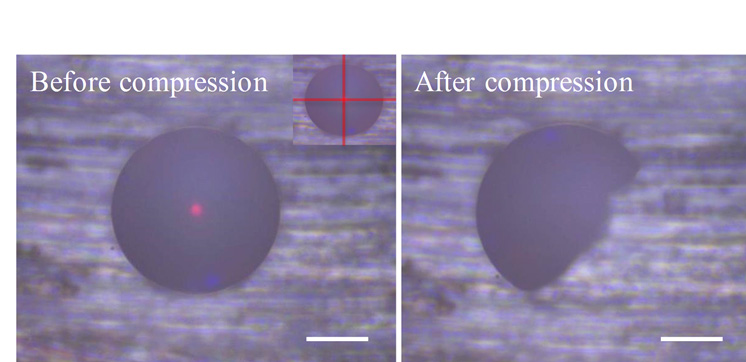


**Figure S8.** Optical images of PDA@SiO_2_ PC beads before and after compression on the NanoTest system. Insert scale bar is 100 µm.


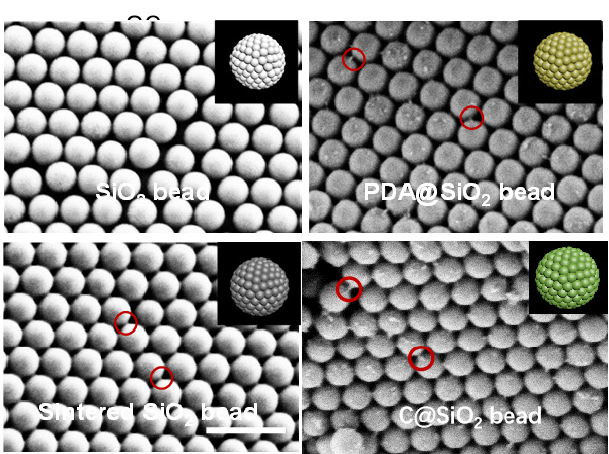


**Figure S9.** Magnified FESEM images of cross-section of the SiO_2_ beads, PDA@SiO_2_ beads,sintered SiO_2_ beads, and C@SiO_2_ beads (the red circles are the adhesive granules). Insert scale bar is 1 µm.

**Figure S10.** Raman spectra of SiO_2_ beads, PDA@SiO_2_ beads, and C@SiO_2_ beads.


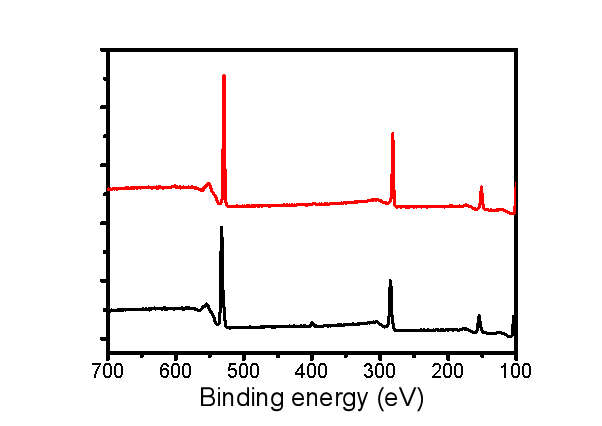


**Figure S11.** XPS spectra of PDA@SiO_2_ PC beads (black) and C@SiO_2_ PC beads (red). XPS analysis reveals a peak corresponding to C1s at 284.8 eV (PDA@SiO_2_: 54.04%, C@SiO_2_: 56.9%), N1s at 401.1 (PDA@SiO_2_: 3.41%, C@SiO_2_: 1.55%) and O1s at 532.1 eV (PDA@SiO_2_: 42.56%, C@SiO_2_: 41.55%).

**Figure S12.** High-resolution XPS narrow scans of the C1s region of PDA (a) and py-PDA (b). The C1s peak reveals C-C at 284.5 eV, C-OH at 285.5 eV, C-N sp2 at 286.1 eV, C-O-C at 286.8 eV, and C N sp3 at 288.2 eV.


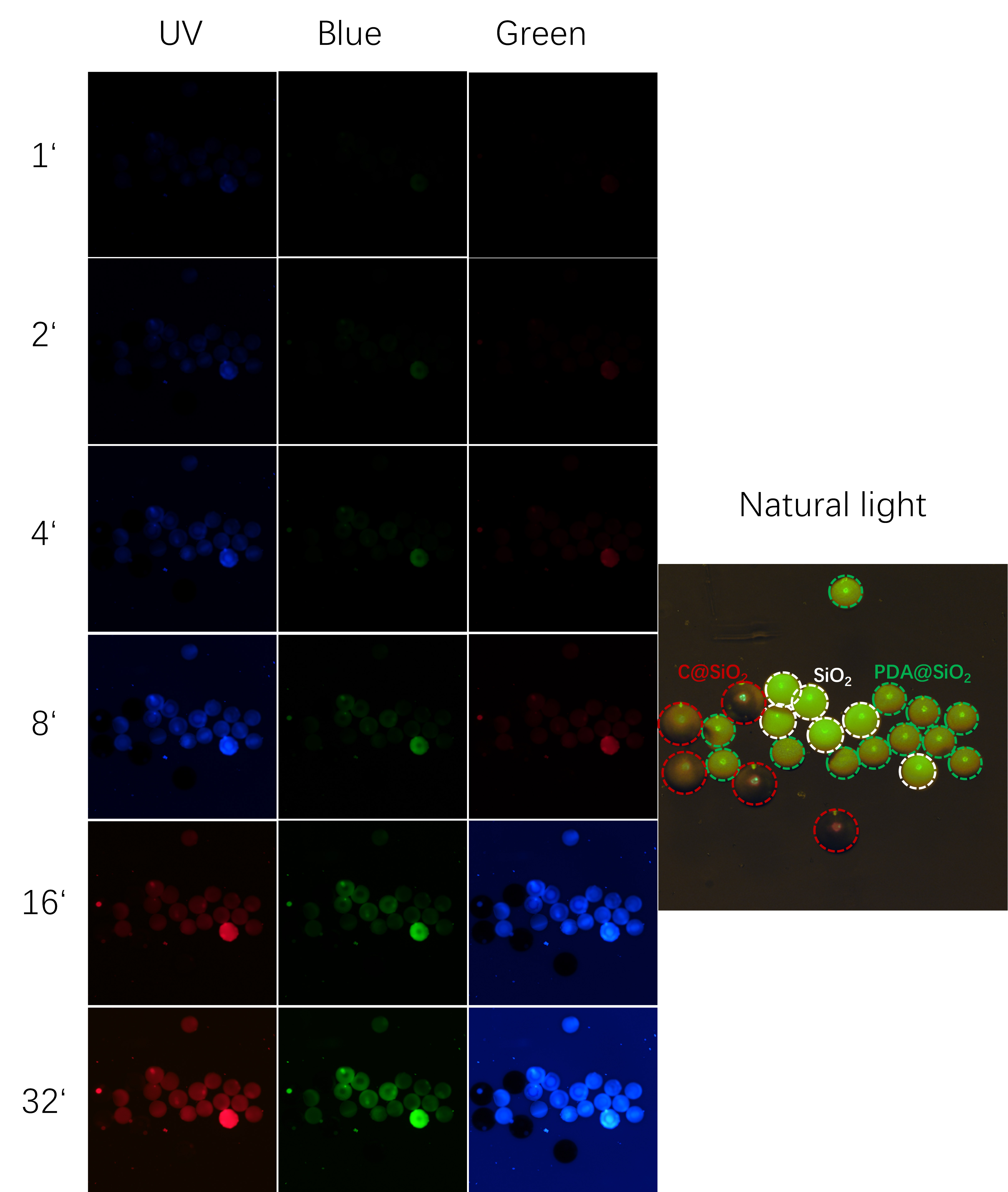


**Figure S13.** The ontology fluorescence intensity of SiO_2_ beads (white circle), PDA@SiO_2_ beads (green circle), and C@SiO_2_ beads (red circle) shoot by UV, blue, green and natural light under exciting light intensity with different degree from 1 to 32.


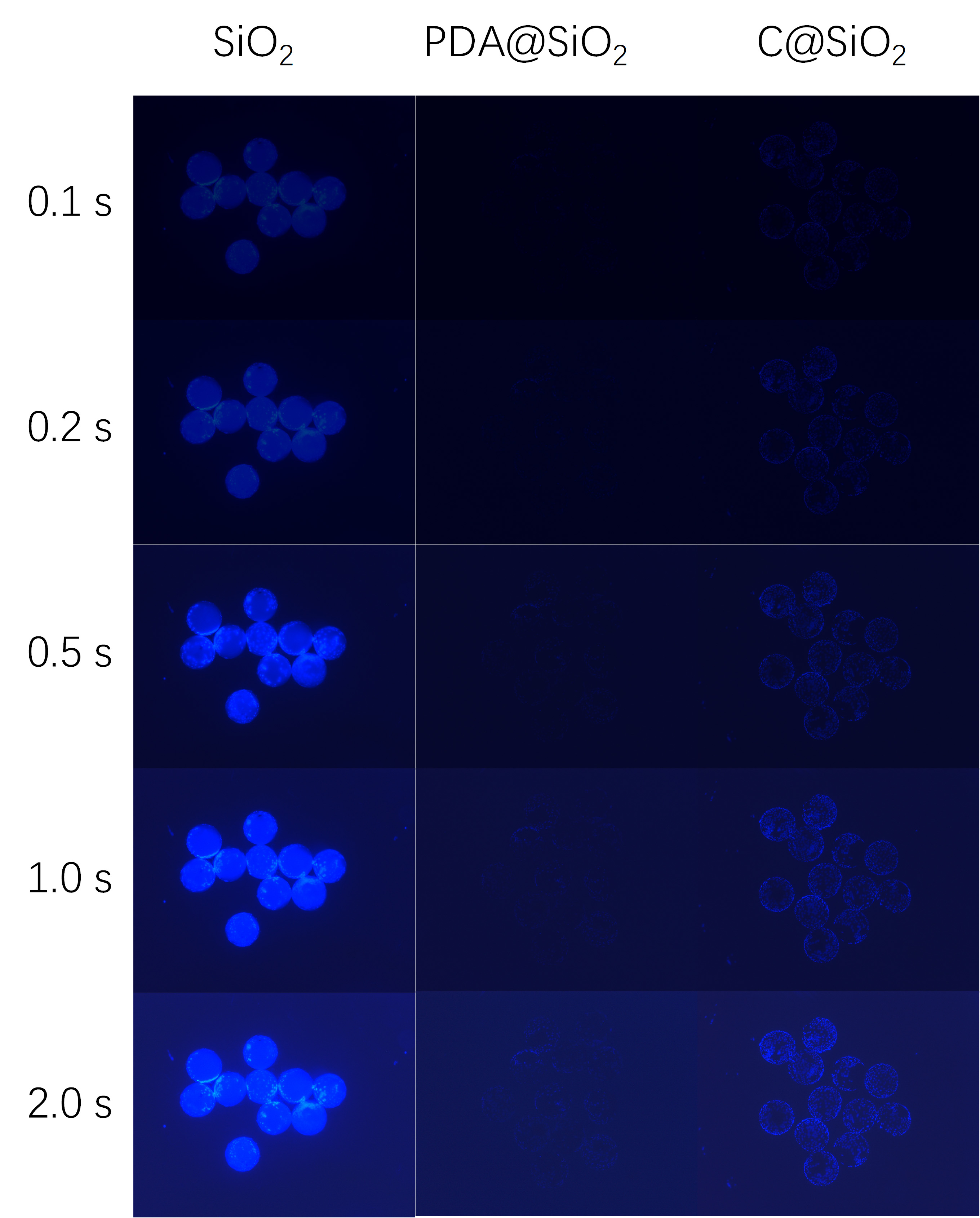


**Figure S14.** Different exposure time obtained fluorescence images of Hoechst 33342 staining cell nucleus of the captured 5-8F cells with the PLL coated sintered SiO_2_ beads, PDA@SiO_2_ beads, and C@SiO_2_ beads, respectively.


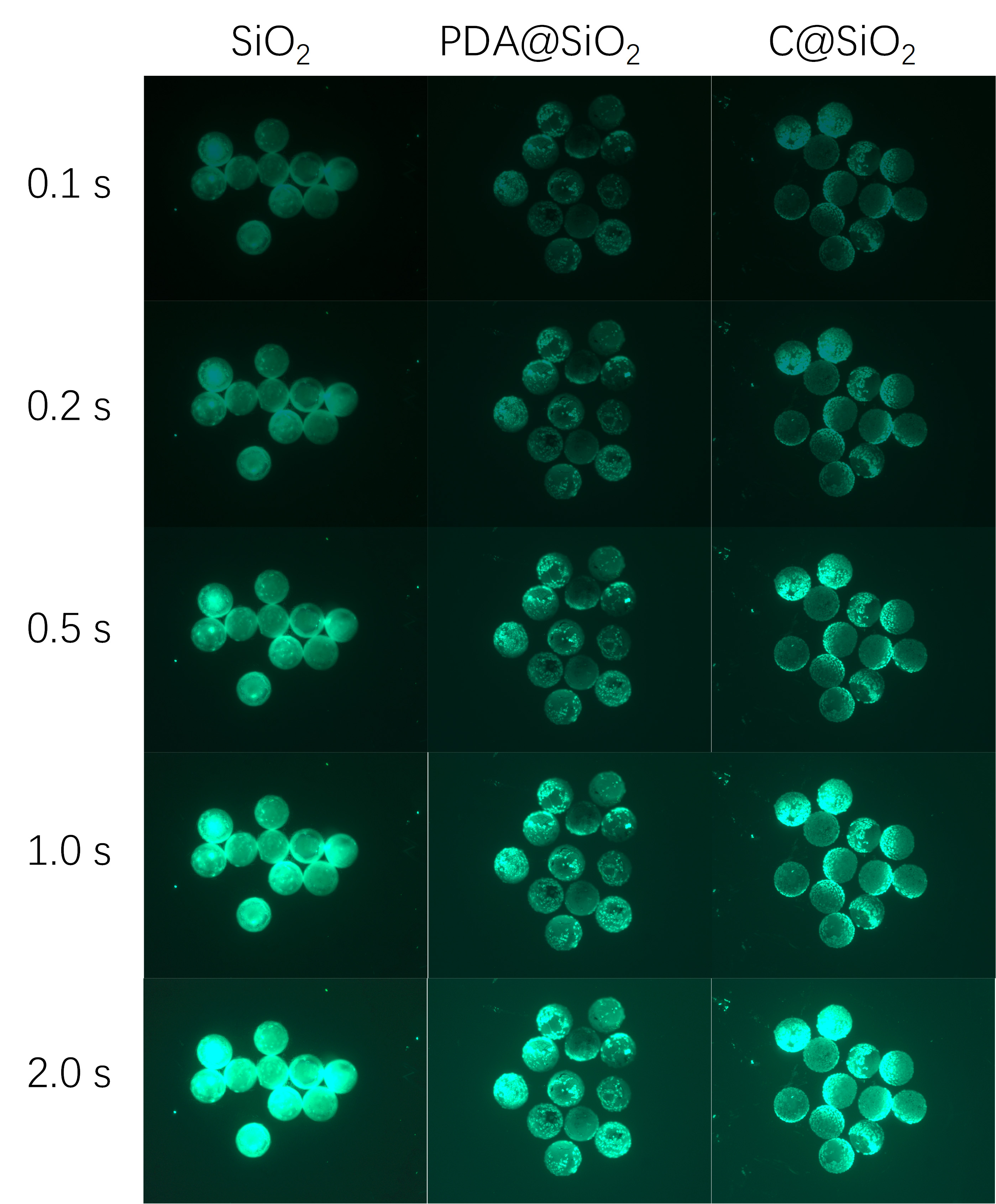


**Figure S15.** Different exposure time obtained fluorescence images of DiO staining cell membrane of the captured 5-8F cells with the PLL coated sintered SiO_2_ beads, PDA@SiO_2_ beads, and C@SiO_2_ beads, respectively.


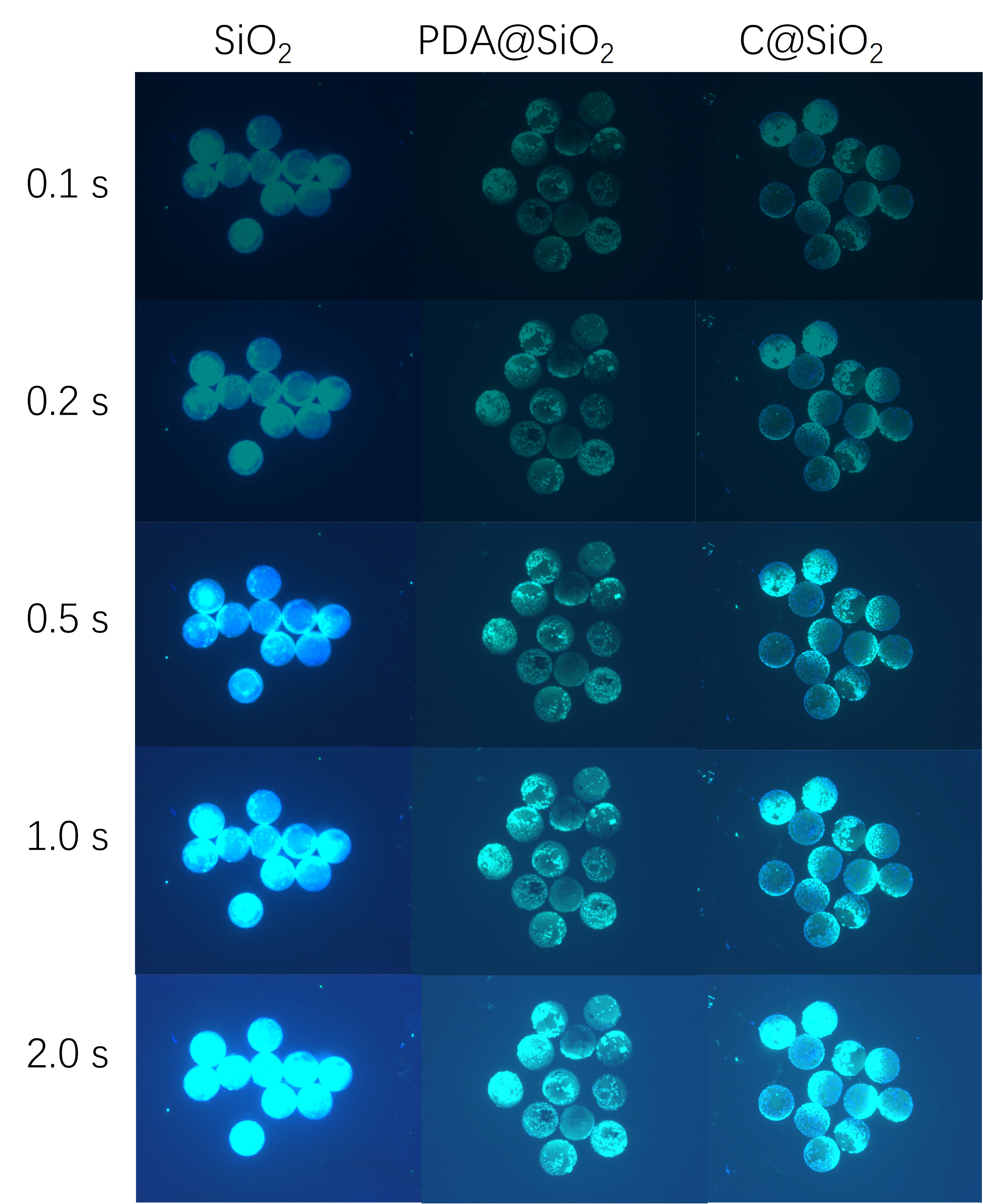


**Figure S16.** Different exposure time obtained fluorescence images of merge images of Hoechst 33342 and DiO (ⅲ) of the captured 5-8F cells with the PLL coated sintered SiO_2_ beads, PDA@SiO_2_ beads, and C@SiO_2_ beads, respectively.

.

**Figure S17.** The ratio of signal-to-noise ratio between C@SiO_2_ barcodes and the sintered SiO_2_ PC barcodes.


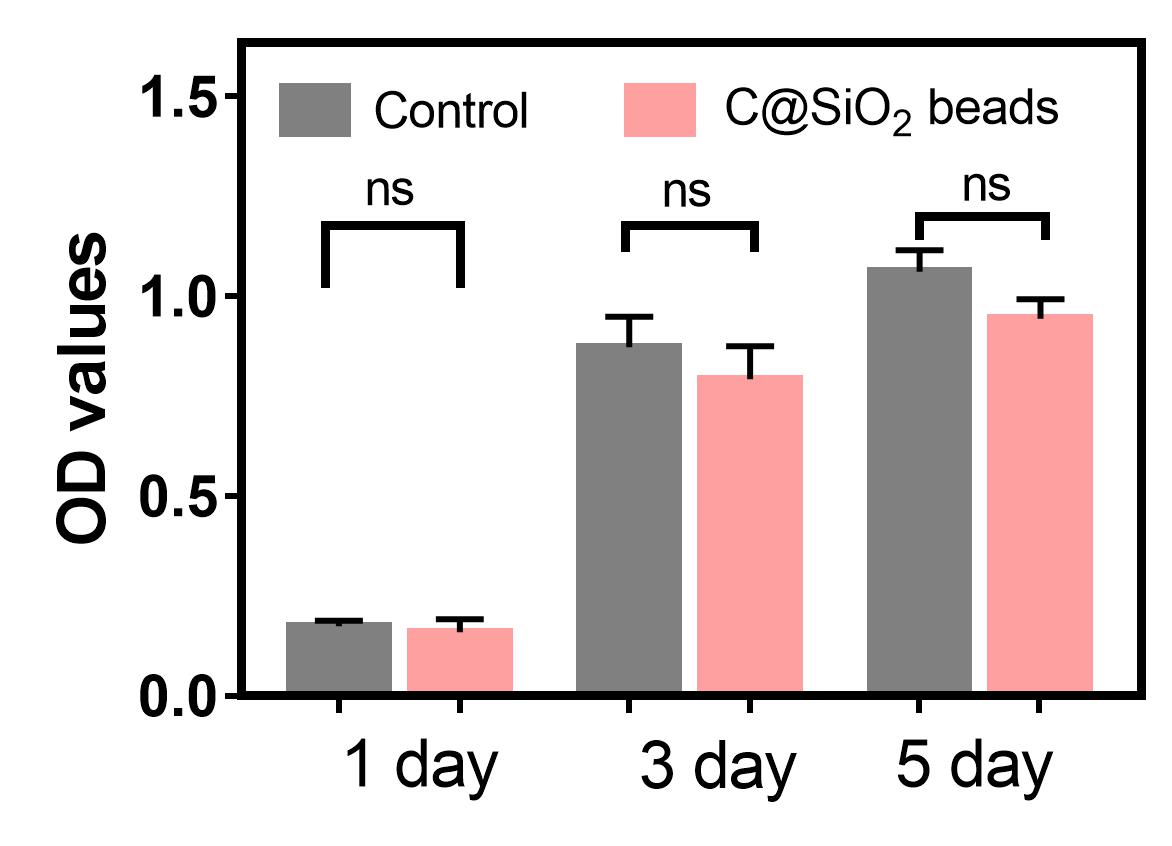


**Figure S18.** CCK-8 results of C@SiO_2_ barcodes culturing with MRC-5 (human embryonic lung fibroblasts).


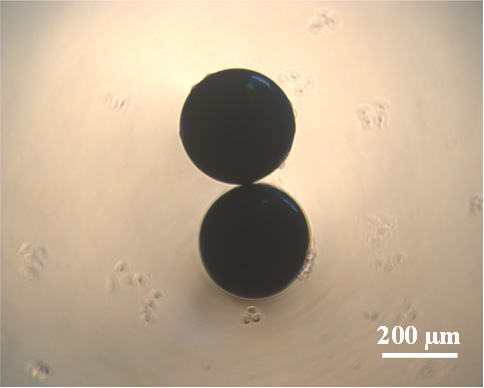


**Figure S19.** Optical microscope photograph of non-PLL coated C@SiO_2_ beads culturing with 5-8F cells on the second day.


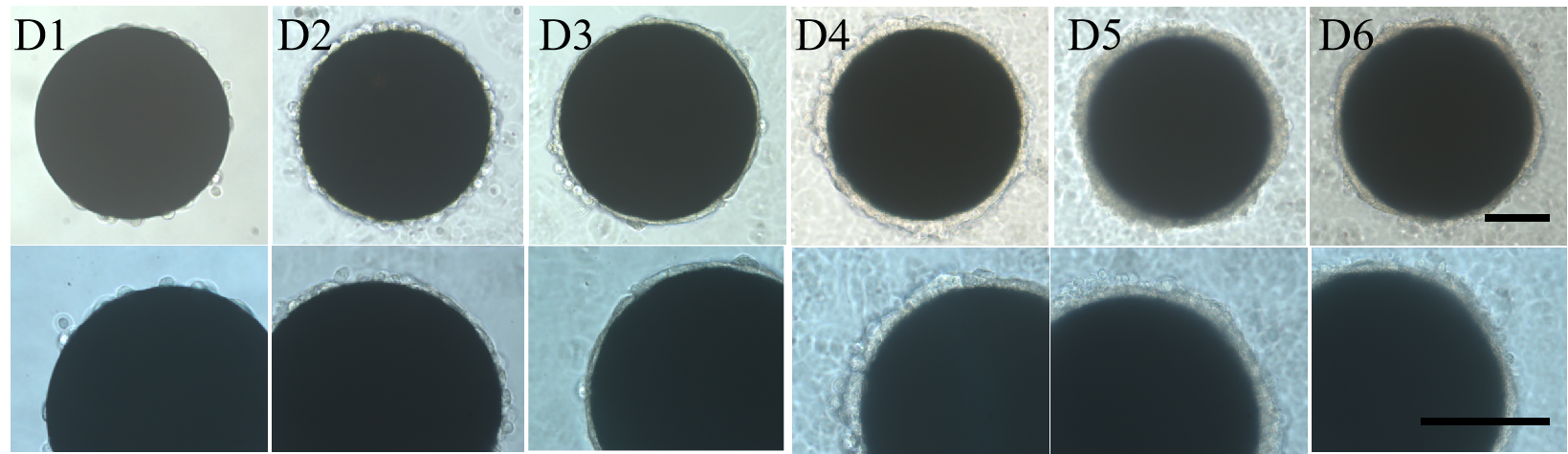


**Figure S20.**  Microscopy images (20×, top row) and zoomed images (40×, bottom row) of PLL coated C@SiO_2_ beads culturing with 5-8F cells from 1 to 6 day. The insert bar is 100 µm.


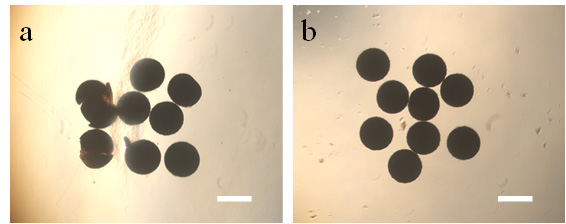


**Figure S21.** Optical microscopy images of PLL coated PDA@SiO_2_ beads (a) and C@SiO_2_ beads(b) culturing with 5-8F cells on the sixth day. Insert scale bar is 300 µm.

**Figure S22.** The fluorescent intensity of background, beads and surface cells in Hoechst 33342, DiO, and merge of Hoechst 33342 and DiO fluorescent-reading images of C@SiO_2_ beads.


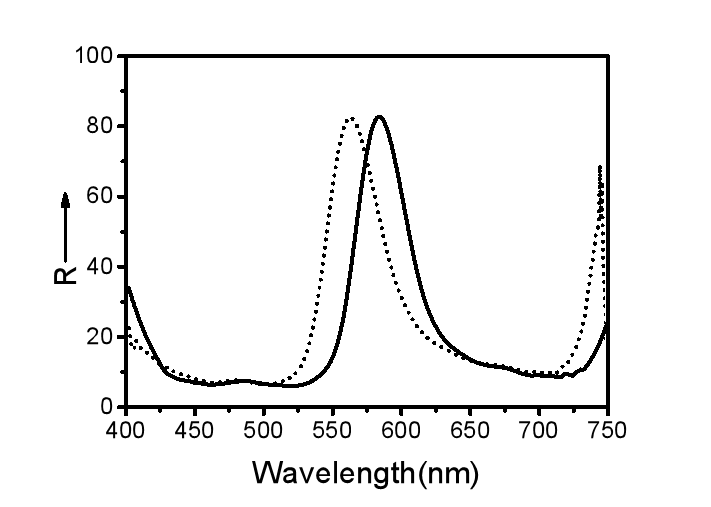


**Figure S23.** The reflectance spectrum of SiO_2_ beads (solid line) and sintered SiO_2_ beads (dotted line).
